# Factors associated with e-cigarette use among vocational students: A cross-sectional multistage cluster survey, Thailand

**DOI:** 10.18332/tid/170421

**Published:** 2023-09-28

**Authors:** Sarunya Benjakul, Saroj Nakju, Lakkhana Termsirikulchai

**Affiliations:** 1 Department of Health Education and Behavioral Sciences, Faculty of Public Health, Mahidol University, Bangkok, Thailand; 2 Faculty of Public Health, Ramkhamhaeng University, Bangkok, Thailand; 3 Tobacco Control Capacity Building Center, Thai Wellbeing Foundation, Bangkok, Thailand

**Keywords:** e-cigarettes use, vocational students, associated factors, PRECEDE framework

## Abstract

**INTRODUCTION:**

The use of e-cigarettes has steadily increased, and vocational students are one primary target of e-cigarette marketing. This cross-sectional survey research aimed to explore e-cigarette use and examine the factors associated with it.

**METHODS:**

Multistage cluster random sampling was employed to select 1536 students in vocational education institutions. A self-administered questionnaire was used to collect the data from September to December 2019. Multinomial logistic regression analysis was used to obtain the adjusted odds ratio (AOR) to determine the associated factors of e-cigarette use.

**RESULTS:**

Altogether, 28.7% of the subjects were currently e-cigarette users, 7.4% used e-cigarettes only, and 21.3% were dual users. Various factors were found to be significantly associated with e-cigarette use by 43.7%. Those consisted of sex (male) (AOR=2.183; 95% CI: 1.510–3.157), grade point average (GPA) of <2.5 (AOR=2.363; 95% CI: 1.502–3.717), having neutral attitudes toward e-cigarette use (AOR=2.676; 95% CI: 1.499–4.779) and positive attitudes toward e-cigarette use (AOR=4.171; 95% CI: 2.250–7.734), moderate level of perceived behavioral control on e-cigarette use (AOR=3.520; 95% CI: 2.287–5.418) and low level of perceived behavioral control on e-cigarettes use (AOR=4.959; 95% CI: 3.274–7.511), moderate price of e-cigarettes (AOR=1.436; 95% CI: 1.009–2.044), and e-cigarette use of their parents (AOR=1.827; 95% CI: 1.137–2.938), close friends (AOR=4.327; 95% CI: 2.954–6.338) and idols (AOR=4.604; 95% CI: 1.844–11.497).

**CONCLUSIONS:**

Students should be encouraged to develop negative attitudes toward e-cigarette use and increase their self-confidence to control the use of e-cigarettes. This can be achieved by regularly distributing information on the product’s risks. Moreover, students can find inspiration and guidance from peers, close friends, or their idols, who will act as positive role models and inspire them not to initiate e-cigarette use.

## INTRODUCTION

Electronic cigarettes (e-cigarettes) are a recent tobacco product widely used among adolescents in the US since 2014^1^. In 2014, 466 brands of e-cigarettes were found on the internet, and 10.5 brands increased monthly^2^. The increased e-cigarette market value was estimated to reach 3.4% annually from 2023 to 2027^3^. E-cigarette packaging has been designed to serve the interests of most users, who are primarily adolescents.

The modern styles and colors of e-cigarette packaging were significantly related to the user’s decision to buy^4,5^. E-cigarettes are offered in various flavors, according to a survey showing that from 2016 to 2017^6^, 15586 tastes and aromas were reported, including a variety of fruit, candy, and bubble gum. The study found that these flavors related to adolescents’ and youths’ beliefs and perceptions that e-cigarettes involve less danger^7-9^. E-cigarettes have reached adolescents extensively and rapidly through different types of social media^10^. Additionally, the survey by Jiang et al.^11^ showed that young adults currently using e-cigarettes have a low level of perceived e-cigarette dangers and a low level of addiction. In contrast, they hold a high level of perception of the modernity of e-cigarettes. Thus, the high spreading rate of e-cigarette use has been found continuously. In 2019, one-tenth (10.5%) of middle school students in the US had used e-cigarettes in the past 30 days, an increase of 17 times compared with the e-cigarette use rate in 2011 (0.6%). For high school students, the percentage of e-cigarette users increased 18 times, from 1.5% to 27.5%, or over one-fourth of high school students using e-cigarettes^12,13^. According to the follow-up study undertaken with senior high school students from 2017 to 2018, the number of e-cigarette users increased 1.7 times, from 11.7% to 20.8%, while among junior high school students, the percentage of e-cigarette users increased 1.5 times, from 3.3% to 4.9%^14^.

In Thailand, adolescents targeted by the e-cigarette business were found to study in general education and vocational education programs with a ratio of 7:3^15^. The related e-cigarette use surveys were conducted only for students in the general education program aged 13–15 years. In 2015, 3.3% of the subjects were current e-cigarette users. Among these users, the number of males was 2.5 times higher than that of females, at 4.7% compared with 1.9%^16^. The literature review of the e-journals in Thailand (Thai Journals Online: ThaiJo) during the past ten years found not only a small number of studies focusing on the target group of vocational students but also addressing traditional tobacco products in this group^17-20^. Related studies showed that students in vocational education programs aged 15–19 years smoked cigarettes at a high rate. According to the studies of Jarujit et al.^18^ and Chomsri et al.^19^, 39.8% and 36.1% were current smokers, respectively. While the national survey in 2017 showed that 10.2% of Thais aged 15–19 years currently smoked^21^.

Therefore, this research aimed to investigate the rate and associated factors of current e-cigarette use among students in vocational institutions. The PRECEDE framework’s third stage, educational and ecological assessment, comprising personal factors, predisposing factors, enabling factors, and reinforcing factors^22^, was applied to comprehensively assess the determining factors influencing e-cigarette use to support tailoring interventions to a specific target group. In addition, the Theory of Planned Behavior (TPB)^23,24^ was applied to explain the predisposing factors regarding attitudes toward e-cigarette use, which are the beliefs driving a person’s behavior, and perceived behavioral control on e-cigarette use, which are the beliefs regarding the ease or difficulty involved in performing the desired behavior. The study findings will be used to develop effective programs to prevent and control adolescent e-cigarette use.

## METHODS

### Research design and participants

The samples for this cross-sectional survey research were the students of vocational schools under the Office of the Vocational Education Commission, Ministry of Education. The sample size was calculated using the estimating and infinite population proportion formulas^25^. In the formula, the proportion of smokers (p) among Thais aged 15–24 years from the national survey in 2017 was 0.218^21^, and the error (d) was 0.05, alpha=0.01, design effect =3.0.

Multistage-cluster random sampling was used to obtain the number of subjects. Nationwide, 875 vocational education institutions have been divided into five strata based on Thailand’s geographical location: North, Northeast, Central, South, and Bangkok. In the first stage, one province was randomly chosen from each stratum, comprising five provinces. For the second stage, a list of vocational education institutes, both government and private, in each province was created. Two institutions were randomly selected from the list, except Bangkok; four institutions were selected. A total of 12 institutions were selected at this stage. In the final stage, five classrooms (one classroom per class year) were randomly selected in each institution, totaling 60 classrooms. All students in each institution meeting the inclusion criteria were included as subjects. The inclusion criteria were: vocational institutions offering study programs for vocational and diploma certificates; institutions with a similar number of students; and institutions with administrators willing to participate in the research project. The inclusion criteria for selecting the sampled students were those attending class on the day of field data collection and those agreeing to provide information by completing the questionnaire.

This research has been approved by the Ethics Committee on Research in Human Beings, Faculty of Public Health, Mahidol University and participants in the research project signed written informed consent forms before data collection. A self-administered questionnaire was used to collect the data from September to December 2019. The subjects were informed of the research objectives and procedures for answering questionnaire items in their classroom. The average time to complete the questionnaires was 20 minutes. Stationery, such as record books and pens, was given as a token of voluntary participation. The questionnaires developed by a researcher aimed to measure the outcome variables and their associated factors as independent variables, as described below.

### Outcome variables

The questions covered current smoked tobacco product use during the past 30 days regarding e-cigarettes, cigarettes, baraka or waterpipe smoking, hand-rolled cigarettes, and other types of smoked tobacco products such as pipes and cigars. Current smoked tobacco use was classified into three groups: 1) non-smokers, those who did not use any smoked tobacco products; 2) current smokers, those who used any smoked tobacco products except e-cigarettes; and 3) current e-cigarette users, those who use e-cigarettes only and dual use. Additionally, another two questions were asked to explore the situation of e-cigarette use regarding methods of obtaining e-cigarettes at the last time of purchase (answers: via online, at a shop, asking someone to buy it for you, requesting for free) and reasons for e-cigarette use (answers: own desire, friends’ persuasion, receiving information of its lower danger, and wanting to quit smoking).

### Independent variables

*Personal factors*

These included sex, age, education level (vocational or diploma certificate), grade point average (GPA) of the last academic semester, monthly income (THB), type of educational institute attended (public or private), and living with parents or guardians (yes or no).

*Predisposing factors*

This part included ten questions concerning knowledge about the dangers of e-cigarettes. For example, nicotine-containing e-cigarettes are less addictive than traditional tobacco products; the aerosol from e-cigarettes is water vapor and does not comprise heavy-metal chemical substances or cancer-causing chemical substances; and an e-cigarette is an alternative for quitting traditionally smoked tobacco products. Each question offered three options: correct, incorrect, and uncertain. a score of 0 (zero) was assigned for the incorrect or uncertain answers, and a score of 1 for the correct answer. Next, attitudes toward e-cigarette use totaled eight items using a seven-point Likert scale from 1 (strongly disagree) to 7 (strongly agree) for positive items and vice versa. For example, e-cigarettes are safer than smoking, e-cigarettes are a fashion trend for adolescents, and e-cigarette use is a common behavior. Then, perceived behavioral control on e-cigarette use comprised six items regarding the degree of people’s confidence to control barriers for performing a given behavior and not using e-cigarettes. For example, ‘confident that you will not use an e-cigarette even if friends are influencing you to’; ‘confident that you will not use an e-cigarette even when receiving information that e-cigarettes are not dangerous’; ‘confident that you will not use an e-cigarette even when you get one for free’, assessed on a seven-point Likert scale from 1 (not strongly confident) to 7 (strongly confident). The summed scores of the three variables, knowledge level about the dangers of e-cigarette use, attitudes toward e-cigarette use, and perceived behavioral control on e-cigarette use, ranged 0–10, 7–56, and 6–48, respectively. These were divided into three groups: low level (60%), moderate level (60 to 79%), and high level (>80%) based on Bloom’s cut-off point^26^.

*Enabling factors*

This part consisted of five questions regarding: 1) being informed about the e-cigarette control laws; for example, educational institutions are smoke-free areas and an e-cigarette is an illegal good according to the declaration of the Ministry of Commerce (yes or no); 2) accessing information about the dangers of an e-cigarette during the past 30 days, for example, by reading news or participating in campaign activities (yes or no); 3) accessing e-cigarette promotion advertisements (yes or no); 4) expressing an opinion about the accessibility of an e-cigarette (easy or difficult); and 5) expressing an opinion on the price of e-cigarettes (cheap, moderate, or expensive). These enabling variables were processed as dummy variables before being analyzed.

*Reinforcing factors*

Three questions determined the e-cigarette use of significant persons, i.e. family members, close friends, and idols such as famous singers, K-pop singer groups, or actors. Each question comprised three answer options: do not use any smoked tobacco products; use any smoked tobacco products except e-cigarettes; and use e-cigarettes. These reinforcing variables were processed in three groups: 1) non-smokers, those who did not use any smoked tobacco products; 2) current smokers, those who used any smoked tobacco products except e-cigarettes; and 3) current e-cigarette users, those who use e-cigarettes only and dual use.

Three experts regarding tobacco control and behavioral science checked the questionnaires for content validity, and 51 college students participated in pretesting the questionnaire. The reliability test results were analyzed using KR-20 and Cronbach’s coefficient alpha. The reliability values concerning knowledge about e-cigarette dangers, attitudes toward e-cigarette use, and perceived behavioral control on e-cigarette use were 0.82, 0.78, and 0.92, respectively.

### Statistical analysis

Data were analyzed by employing SPSS, Version 18.0, copyrighted by Mahidol University. Descriptive statistics were used to assess the sample characteristics. Chi-squared tests with p<0.05 as a significance level were used to compare differences in frequency distributions between groups of smokers and general characteristics. Multinomial logistic regression investigated the associated factors with current e-cigarette use. The significant variables identified by bivariate analysis with p<0.25 were included in the multivariate analysis. The strength of the association was indicated as an adjusted odds ratio (AOR) with 95% confidence interval (CI). To ensure statistical significance, a two-tailed test was employed at a threshold of 0.05, and the likelihood ratio tests determined the goodness of fit of the model (Supplementary file).

## RESULTS

### Sample characteristics

In the sample of 1536 students, males and females were in similar proportions, at 58.6% and 41.4%, respectively, with an average age of 18.1 years (SD=1.7), studying for vocational certificates, 67.2%, and private vocational institutions, 53.3%. The subjects obtained a GPA of 3.1 (SD=0.6) for the last academic semester. They received an average of 4375.7 THB (1000 Thai Baht about US$28) per month (SD=2912.9). Almost all (94.8%) received their expenses from their guardians, and 82.5% lived with their guardians.

### Current e-cigarette use

**Figure 1:**
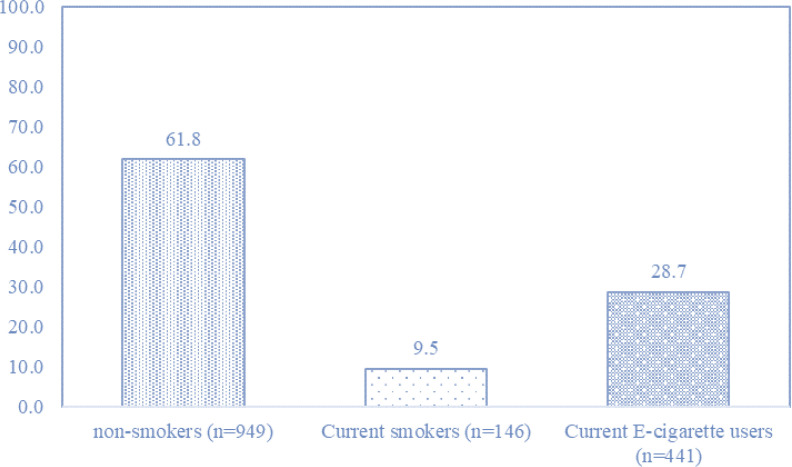
Percentage of current smoke tobacco use among vocational students (N=1536)

The 1536 sampled students were divided into three groups based on whether or not they had used e-cigarettes over the past 30 days. group 1, non-smokers (61.8%: 95% CI: 59.4–64.2); group 2, current smokers (9.5%; 95% CI: 8.0–10.9); and group 3, current e-cigarette users (28.7%; 95% CI: 26.5–31.0). In group 3, 7.4% used only e-cigarettes, while 21.3% were dual users (Figure 1). Moreover, 47.6% of current e-cigarette users received their latest e-cigarettes by asking someone to buy an e-cigarette for them. Further, 29.9% and 14.1% bought online and from the shops, respectively. The most common reason for e-cigarette use was desire (42.2%). According to Table 1, four of seven variables regarding general characteristics indicated a statistically significant difference in the type of smoker. These included sex (p<0.001), type of institute (p<0.001), GPA of last semester (p<0.001), and monthly income (p<0.020).

**Table 1:** General characteristics by type of smoker

*Characteristics*	*Total*	*Non-smokers (group 1)*		*Current smokers (group 2)*		*Current e-cigarette users (group 3)*		*p*
	*n*	*n*	*%*	*n*	*%*	*n*	*%*	
**Sex** (n=1536)								**<0.001**
Female	636	492	77.4	35	5.5	109	17.1	
Male	900	457	50.8	111	12.3	332	36.9	
**Age** (years) (n=1432)								0.093
<18	733	473	64.5	52	7.1	208	28.4	
18–19	491	295	60.1	54	11.0	142	28.9	
≥20	208	121	58.2	23	11.1	64	30.8	
**Current year class** (n=1534)								0.605
Vocational certificate	1031	630	61.1	97	9.4	304	29.5	
Diploma certificate	503	319	63.4	48	9.5	136	27.0	
**Type of institute** (n=1536)								**<0.001**
Public	718	522	72.7	45	6.3	151	21.0	
Private	818	427	52.2	101	12.3	290	35.5	
**GPA of last semester** (n=1425)								**<0.001**
<2.5	212	94	44.3	28	13.2	90	42.5	
≥2.5	1213	793	65.4	105	8.6	315	26.0	
**Monthly income** (THB) (n=1324)								**0.020**
≤3000	604	396	65.6	50	8.3	158	26.2	
>3000–6000	549	324	59.0	63	11.5	162	29.5	
>6000	171	96	56.1	13	7.6	62	36.3	
**Living with parents/guardians** (n=1522)								0.879
No	266	162	60.9	27	10.2	77	28.9	
Yes	1256	784	62.4	118	9.4	354	28.2	

THB: 1000 Thai Baht about US$28.

### Factors associated with current e-cigarette use

According to the results in Table 2, multiple factors were significantly associated with e-cigarette use based on the crude odds ratio (OR) from the bivariate analysis. After employing the AOR using multivariate analysis, ten statistically significant factors were associated with e-cigarette use, accounting for 43.7% (Nagelkerke R^2^=0.437). These factors comprised: 1) personal factors, such as sex and GPA; 2) predisposing factors, such as moderate and positive attitudes toward e-cigarette use, and moderate and low levels of perceived behavioral control on e-cigarette use; 3) enabling factors regarding having perceived the moderate price of the e-cigarette; and 4) reinforcing factors, such as the e-cigarette use of their parents, close friends, and idols.

**Table 2:** Multinomial logistic regression analysis to investigate the associated factors with e-cigarette use among the sampled vocational students

*Factors*	*OR (95% CI)*	*p*	*AOR (95% CI)*	*p*
**Personal factors**				
**Sex**				
Female (Ref.)	1		1	
Male	3.279 (2.551–4.215)	**<0.001**	2.183 (1.510–3.157)	**<0.001**
**Age** (years)				
<18 (Ref.)	1			
18–19	1.095 (0.846–1.417)	0.493		
≥20	1.203 (0.853–1.697)	0.293		
**Current year class**				
Vocational certificate (Ref.)	1			
Diploma certificate	0.884 (0.693–1.126)	0.318		
**Type of institution**				
Public (Ref.)	1		1	
Private	2.348 (1.857–2.969)	**<0.001**	1.134 (0.801–1.606)	0.478
**Grade point average** (GPA)				
≥2.5 (Ref.)	1		1	
<2.5	2.410 (1.755–3.310)	**<0.001**	2.363 (1.502–3.717)	**<0.001**
**Monthly income** (THB)				
≤3000 (Ref.)	1		1	
>3000–6000	1.253 (0.963–1.631)	0.094		
>6000	1.619 (1.119–2.341)	**0.010**	1.169 (0.689–1.984)	0.562
**Living with parents/guardians**				
Yes (Ref.)	1			
No	1.053 (0.781–1.419)	0.736		
**Predisposing factors**				
**Knowledge about dangers of e-cigarettes**				
High (Ref.)	1		1	
Moderate	2.185 (1.180–4.043)	**0.013**	1.426 (0.555–3.664)	0.461
Low	1.306 (0.644–2.648)	0.458		
**Attitude toward e-cigarette use**				
Negative (Ref.)	1		1	
Neutral	6.196 (3.916–9.804)	**<0.001**	2.676 (1.499–4.779)	**0.001**
Positive	17.640 (11.124–27.971)	**<0.001**	4.171 (2.250–7.734)	**<0.001**
**Perceived behavioral control on e-cigarette use**				
High (Ref.)	1		1	
Moderate	6.630 (4.768–9.219)	**<0.001**	3.520 (2.287–5.418)	**<0.001**
Low	11.308 (8.390–15.241)	**<0.001**	4.959 (3.274–7.511)	**<0.001**
**Enabling factors**				
**Be informed about e-cigarette control laws**				
Yes (Ref.)	1		1	
No	2.506 (1.882–3.338)	**<0.001**	1.306 (0.863–1.976)	0.207
**Access to information or participating in activities about dangers of e-cigarettes**				
Yes (Ref.)	1			
No	0.825 (0.656–1.036)	0.098		
**Access to e-cigarette advertising and promotions through social media**				
No (Ref.)	1		1	
Yes	1.689 (1.343–2.124)	**<0.001**	1.226 (0.874–1.720)	0.239
**Opinion on being easy or difficult to buy e-cigarettes**				
Difficult (Ref.)	1			
Easy	1.169 (0.854–1.599)	0.330		
**Opinion on the price of e-cigarettes**				
Expensive (Ref.)	1		1	
Moderate	2.124 (1.636–2.758)	**<0.001**	1.436 (1.009–2.044)	**0.045**
Cheap	1.906 (1.208–3.008)	**<0.001**	1.573 (0.842–2.939)	0.156
**Reinforcing factors**				
**E-cigarette use of family members**				
Non-smokers (Ref.)	1		1	
Smokers	1.129 (0.876–1.456)	0.348		
E-cigarette users	1.991 (1.445–2.744)	**<0.001**	1.827 (1.137–2.938)	**0.013**
**E-cigarette use of close friends**				
Non-smokers (Ref.)	1		1	
Smokers	4.838 (3.384–6.917)	**<0.001**	3.055 (1.898–4.917)	**<0.001**
E-cigarette users	8.015 (5.993–10.720)	**<0.001**	4.327 (2.954–6.338)	**<0.001**
**E-cigarette use of favorite persons** (idols)				
Non-smokers (Ref.)	1		1	
Smokers	1.300 (0.770–2.197)	0.327		
E-cigarette users	7.164 (3.592–14.287)	**<0.001**	4.604 (1.844–11.497)	**0.001**
	Nagelkerke R^2^=0.437 , Intercept= -5.009, p <0.001			

AOR: adjusted odds ratio. The table displays statistical results obtained from multinomial logistic regression, comparing group 3 (current e-cigarette users) with group 1 (non-smokers), in line with the study’s objective. THB: 1000 Thai Baht about US$28.

## DISCUSSION

This study showed that 3 in 10 sampled students currently used e-cigarettes. The rate was higher than other studies conducted over a similar time frame; 3.3% of students aged 13–15 years enrolling in general education schools were found to use e-cigarettes in 2017^16^. According to the National Health Examination Survey (NHES), from 2019 to 2020, 2.9% of Thais aged 10–19 years currently used e-cigarettes^27^. A similar finding was reported in the study about students’ e-cigarette use in different vocational education schemes^28^. This finding of a higher percentage of vocational students using e-cigarettes could be because the vocational education program aims to enhance students’ vocational skills. Therefore, they must practice in the actual situation, act as adults, and work closely with the working-age group. They may become stressed due to the mentioned activities of studying and practicing during the same period. These environments are conducive to smoking.

Many factors associated with e-cigarette use were found in this study. Studies have shown that males are 2.2 times more likely to use e-cigarettes than females. This finding was consistent with related studies^29-31^. Regarding educational achievement (GPA), subjects with a GPA <2.5 were 2.4 times more likely to use e-cigarettes than those with a GPA ≥2.5. Therefore, various smoked tobacco products, including e-cigarettes, were their choices because every smoked tobacco product contains nicotine that simulates dopamine release. Dopamine is strongly associated with a sense of pleasure, happiness, and decreased stress and anxiety^32^. The relationship between smoking and educational achievement was also found in related studies^33^.

Positive attitudes toward e-cigarette use were significantly associated with e-cigarette use due to attitudes concerning the person’s thoughts, beliefs, or feelings that can encourage action. Individual attitudes can typically be developed based on their knowledge and impression of the received information or message^34^. E-cigarette products’ packaging, flavors, and advertising on various social media platforms might persuade clients to gain positive attitudes or believe e-cigarettes provide less harmful effects^35^. Perceived behavioral control on e-cigarette use involves the person’s self-confidence. Whether or not it would be easy or difficult to control themselves to perform behaviors even though that person is facing a complex or challenging situation would link directly to the action, especially in a situation with adequate information related to that action^36^. According to related studies, subjects showed less perceived behavioral control on e-cigarette use, which resulted in a higher chance of using e-cigarettes. Moreover, related studies found a relationship between advertisements, the decision to try, and e-cigarette use^37^. Therefore, access to e-cigarette advertisements through various channels, mainly social media, could develop impressions and positive attitudes toward e-cigarette use, decreasing perceived behavioral control over e-cigarette use and influencing decisions to use e-cigarettes.

Based on the study’s findings, those in the sample who thought e-cigarettes were moderately priced were 1.3 times more likely to use them compared with those who thought they were expensive. The decision to buy any product depends on the product’s price, which varies in the same direction as the product’s quality^38^. Therefore, for products with a high cost, even though consumers do not have purchasing power, they perceive that the product possesses high quality. However, for products priced lower, the consumer will perceive that the product possesses low quality. Lastly, the product with a moderate or reasonable price will become the consumer’s choice. The reinforcing factors in the PRECEDE framework focus on the wide range of external factors influencing positive and negative desired behaviors, such as social support, incentives, rewards, positive feedback, or role models^22^. On the other hand, subjective norms, a crucial construct in the Theory of Planned Behavior^24^, reflect an individual’s perception of the social pressure or expectations from significant others regarding specific behaviors. Therefore, the study revealed that the students’ influencers using e-cigarettes – their parents, close friends, and idols – significantly increased the chance that the sample used e-cigarettes by 1.8 to 4.6 times.

### Strengths and limitations

The strength of this study was that the subjects were selected using multistage cluster random sampling and probability sampling. The questionnaire was valid and reliable, and the comprehensive method could serve as valuable learning for others. One limitation was the process of data collection using paper and pencil for the administration of the questionnaire. Even though the informants had received privacy protection by not identifying names and addresses and had been assured the data collected were confidential, following ethical research principles among human subjects, incomplete data occurred for many reasons. For example, individuals did not answer some items, skipped reading, or did not remember. However, only a little incomplete information was found because the questionnaires had been checked for completeness before being returned from the study field. Another limitation was the research design, a cross-sectional study that could not establish a cause-and-effect relationship or analyze e-cigarette use over time. A cohort study is recommended to address this issue.

## CONCLUSIONS

The factors associated with using e-cigarettes need to be considered when designing school-based interventions to prevent e-cigarette use. In addition, supportive environments encouraging happy living should be created outside classrooms or during class periods when no instruction is provided to motivate students with low academic achievement (GPA), lessen boredom and stress, and kept at school happily, which can also prevent initial e-cigarette use.

## Data Availability

The data supporting this research cannot be made available for privacy or other reasons.

## References

[1] US Department of Health and Human Services (2016). E-Cigarette Use Among Youth and Young Adults. A Report of the Surgeon General.

[2] Zhu SH, Sun JY, Bonnevie E (2014). Four hundred and sixty brands of e-cigarettes and counting: implications for product regulation. Tob Control.

[3] (2023). E-Cigarettes - Worldwide.

[4] Ahmed RR, Parmar V, Ahmed MA (2014). Impact of product packaging on consumer’s buying behavior. European Journal of Scientific Research.

[5] Chitroda J, Patel P (2020). A study on product packaging impact on consumer buying behaviour. International Journal of Novel Research in Marketing Management and Economics.

[6] Hsu G, Sun JY, Zhu SH (2018). Evolution of electronic cigarette brands from 2013-2014 to 2016-2017: analysis of brand websites. J Med Internet Res.

[7] Pepper JK, Ribisl KM, Brewer NT (2016). Adolescents’ interest in trying flavoured e-cigarettes. Tob Control.

[8] Zare S, Nemati M, Zheng Y (2018). A systematic review of consumer preference for e-cigarette attributes: flavor, nicotine strength, and type. PLoS One.

[9] Strombotne K, Buckell J, Sindelar JL (2021). Do JUUL and e-cigarette flavours change risk perceptions of adolescents? Evidence from a national survey. Tob Control.

[10] Murukutla N, Magsumbol MS, Raskin H (2022). A content analysis of e-cigarette marketing on social media: findings from the Tobacco Enforcement and Reporting Movement (TERM) in India, Indonesia and Mexico. Front Public Health.

[11] Jiang N, Cleland CM, Wang MP, Kwong A, Lai V, Lam TH (2019). Perceptions and use of e-cigarettes among young adults in Hong Kong. BMC Public Health.

[12] Corey CG, Ambrose BK, Apelberg BJ, King BA (2015). Flavored Tobacco Product Use Among Middle and High School Students--United States, 2014. MMWR Morb Mortal Wkly Rep.

[13] Cullen KA, Gentzke AS, Sawdey MD (2019). E-cigarette use among youth in the United States, 2019. JAMA.

[14] Centers for Disease Control and Prevention Tobacco Use By Youth Is Rising: E-cigarettes are the main reason.

[15] Information Technology & Communication Center, Ministry of Education (2021). Proportion of Students in Upper Secondary Level General Education Type to Vocational Education Type: Academic Year 2017-2021.

[16] Chotbenjamaporn P, Haruhansapong V, Jumriangrit P, Pitayarangsarit S, Agarwal N, Garg R (2017). Tobacco use among thai students: results from the 2015 global youth tobacco survey. Indian J Public Health.

[17] Veerasuksawatt R, Prakenree N, Duagmala W, Thongnun W, Jaisupap P, Trychasirikosol T (2013). The prevalence of smoking behaviour and factors related to smoking behaviour among female students of high schools and vocational colleges in North-Eastern Thailand. Article in Thai. Sanpasitthiprasong Medical Journal.

[18] Jarujit S, Srisuriyawet R, Homsin P (2015). Factors associated with regular smoking among male vocational students in Chanthaburi Province. Article in Thai. Journal of Nursing and Education.

[19] Chomsri P, Aramratana A, Siviroj P, Kuntawee S (2017). Prevalence of substance use, and association between substance use and sensation seeking among vocational students. Article in Thai. Nursing Journal.

[20] Mahasuan S, Homsin P, Srisuriyawet R (2018). Factors related to smoking initiation among female vocational students in Chachoengsao Province. Article in Thai. Journal of Public Health Nursing.

[21] National Statistical Office of Thailand (2018). The smoking and drinking behaviour survey 2017.

[22] Gielen AC, McDonald EM, Gary TL, Bone LR, Glanz K, Rimer BK, Viswanath K (2008). Using the PRECEDE-PROCEED model to apply health behavior theories. Health behavior and health education: Τheory, Research, and Practice.

[23] Ajzen I, Kuhl J, Beckmann J (1985). From intention to actions: A theory of planned behavior. Action control: From cognition to behavior.

[24] Ajzen I (2002). Perceived Behavioral Control, Self-Efficacy, Locus of Control, and the Theory of Planned Behavior. J Appl Soc Psychol.

[25] Wayne WD (1995). Biostatistics: A Foundation for Analysis in the Health Sciences.

[26] Bloom BS, Madaus GF, Hastings JT (1971). Handbook on Formative and Summative Evaluation of Student Learning.

[27] Patanavanich R, Vityananan P, Neelapaichit N (2022). Association between electronic cigarette use and depression among Thai adolescents: The Thailand National Health Examination Survey 2019-2020. Tob Induc Dis.

[28] Zhu J, Li J, Xu G, Yu J, Wang Q, He Y (2020). School-type differences in e-cigarette use and its correlates among Chinese adolescents. Tob Induc Dis.

[29] Rodríguez-Bolaños R, Arillo-Santillán E, Barrientos-Gutiérrez I, Zavala-Arciniega L, Ntansah CA, Thrasher JF (2019). Sex differences in becoming a current electronic cigarette user, current smoker and current dual user of both products: a longitudinal study among Mexican adolescents. Int J Environ Res Public Health.

[30] Irvine DS, Lee EY, Janssen I, Leatherdale ST. (2021). Gendered associations between e-cigarette use, cigarette smoking, physical activity, and sedentary behaviour among Canadian adolescents. Paper presented at: Proceedings from the 8th International Society for Physical Activity and Health Congress. The Health & Fitness Journal of Canada.

[31] AlMuhaissen S, Mohammad H, Dabobash A, Nada MQ, Suleiman ZM (2022). Prevalence,knowledge, and attitudes among health professions students toward the use of electronic cigarettes. Healthcare (Basel).

[32] Benowitz NL (2009). Pharmacology of nicotine: addiction, smoking-induced disease, and therapeutics. Annu Rev Pharmacol Toxicol.

[33] Ullah S, Sikander S, Abbasi MMJ (journal). Association between smoking and academic performance among undergraduate students of Pakistan, A cross-sectional study. Research Square. Preprint posted online August.

[34] Ajzen I, Fishbein M (2000). Attitudes and the attitude-behavior relation: Reasoned and automatic processes. Eur Rev Soc Psychol.

[35] Hung M, Spencer A, Goh C (2022). The association of adolescent e-cigarette harm perception to advertising exposure and marketing type. Arch Public Health.

[36] Ajzen I (1991). The theory of planned behavior. Organ Behav Hum Decis Process.

[37] Padon AA, Lochbuehler K, Maloney EK, Cappella JN (2018). A randomized trial of the effect of youth appealing e-cigarette advertising on susceptibility to use e-cigarettes among youth. Nicotine Tob Res.

[38] Judd VC (2000). The price-quality relationship: an empirical study of food products. J Food Prod Mark.

