# Supplementary material for: Factors associated with e-cigarette use among vocational students: A cross-sectional multistage cluster survey, Thailand
Source: Tob Induc Dis. 2023 Sep 28;21:120. doi: 10.18332/tid/170421 (PMC10535840; doi:10.18332/tid/170421)
Supplement: Supplementary file 1 [file TID-21-120-s1.pdf]

Model Fitting Information

Model	Model Fitting Criteria	Likelihood Ratio Tests		
	-2 Log Likelihood	Chi-Square	df	Sig.
Intercept Only	1976.856			
Final	1481.237	495.619	42	.000

Pseudo R-Square

Cox and Snell	.371
Nagelkerke	.437
McFadden	.245

Likelihood Ratio Tests

Effect	Model Fitting Criteria	Likelihood Ratio Tests		
	-2 Log Likelihood of Reduced Model	Chi-Square	df	Sig.
Intercept	1620.667	139.429	2	.000
Gender	1501.128	19.890	2	.000
Owner_institute	1489.289	8.052	2	.018
GRADE_2GRs	1496.429	15.192	2	.001
Dummy_Income3to6000	1483.021	1.784	2	.410
Dummy_IncomeOver6000	1482.501	1.264	2	.532
Dummy_K_Middle	1481.847	.610	2	.737
Dummy_K_Low	1482.915	1.678	2	.432
Dummy_ATT_Middle	1493.695	12.458	2	.002
Dummy_ATT_GoodtoEcig	1503.987	22.749	2	.000
Dummy_SE_Middle	1516.424	35.187	2	.000
Dummy_SE_Bad	1541.123	59.886	2	.000
EnableNew	1482.856	1.619	2	.445
Received_TAPsEcig	1482.631	1.394	2	.498
Dummy_EN9_Middle	1485.390	4.153	2	.125
Dummy_EN9_Cheap	1483.273	2.036	2	.361
Dummy_HH_Ecig	1487.760	6.523	2	.038
Dummy_HH_SMnotEcig	1487.460	6.223	2	.045
Dummy_friend_Ecig	1541.107	59.870	2	.000
Dummy_friend_SMnotEcig	1512.648	31.410	2	.000
Dummy_IDOL_Ecig	1493.777	12.540	2	.002
Dummy_IDOL_SMnotEcig	1483.786	2.549	2	.280

The chi-square statistic is the difference in -2 log-likelihoods between the final model and a reduced model. The reduced model is formed by omitting an effect from the final model. The null hypothesis is that all parameters of that effect are 0.

Parameter Estimates

จัดกลุ่มการใช้ ecig และบุหรี่อื่นๆ[a]		B	Std. Error	Wald	df	Sig.	Exp(B)	95% Confidence Interval for Exp(B)	
								Lower Bound	Upper Bound
ใช้/สูบ eCig ปัจจุบัน	Intercept	-5.009	.535	87.769	1	.000			
	Gender	.781	.188	17.235	1	.000	2.183	1.510	3.157
	Owner_institute	.126	.178	.503	1	.478	1.134	.801	1.606
	GRADE_2GRs	.860	.231	13.838	1	.000	2.363	1.502	3.717
	Dummy_Income3to6000	.151	.183	.681	1	.409	1.163	.812	1.666
	Dummy_IncomeOver6000	.157	.270	.337	1	.562	1.169	.689	1.984
	Dummy_K_Middle	.355	.481	.544	1	.461	1.426	.555	3.664
	Dummy_K_Low	.472	.414	1.298	1	.255	1.603	.712	3.608
	Dummy_ATT_Middle	.984	.296	11.081	1	.001	2.676	1.499	4.779
	Dummy_ATT_GoodtoEcig	1.428	.315	20.555	1	.000	4.171	2.250	7.734
	Dummy_SE_Middle	1.258	.220	32.690	1	.000	3.520	2.287	5.418
	Dummy_SE_Bad	1.601	.212	57.120	1	.000	4.959	3.274	7.511
	EnableNew	.267	.211	1.596	1	.207	1.306	.863	1.976
	Received_TAPsEcig	.204	.173	1.387	1	.239	1.226	.874	1.720
	Dummy_EN9_Middle	.362	.180	4.031	1	.045	1.436	1.009	2.044
	Dummy_EN9_Cheap	.453	.319	2.014	1	.156	1.573	.842	2.939
	Dummy_HH_Ecig	.603	.242	6.191	1	.013	1.827	1.137	2.938
	Dummy_HH_SMnotEcig	.324	.190	2.919	1	.088	1.383	.953	2.005
	Dummy_friend_Ecig	1.465	.195	56.555	1	.000	4.327	2.954	6.338
	Dummy_friend_SMnotEcig	1.117	.243	21.161	1	.000	3.055	1.898	4.917
	Dummy_IDOL_Ecig	1.527	.467	10.693	1	.001	4.604	1.844	11.497
	Dummy_IDOL_SMnotEcig	-.536	.377	2.021	1	.155	.585	.279	1.225

ใช้/สูบปัจจุบัน แต่ไม่ใช่ eCig	Intercept	-4.382	.613	51.057	1	.000			
	Gender	.691	.253	7.456	1	.006	1.996	1.215	3.277
	Owner_institute	.671	.242	7.695	1	.006	1.956	1.218	3.143
	GRADE_2GRs	.746	.290	6.616	1	.010	2.108	1.194	3.721
	Dummy_Income3to6000	.299	.232	1.659	1	.198	1.348	.856	2.125
	Dummy_IncomeOver6000	-.259	.392	.437	1	.509	.772	.358	1.665
	Dummy_K_Middle	.254	.552	.212	1	.645	1.290	.437	3.808
	Dummy_K_Low	-.103	.490	.044	1	.834	.902	.346	2.357
	Dummy_ATT_Middle	.389	.318	1.499	1	.221	1.476	.791	2.752
	Dummy_ATT_GoodtoEcig	.541	.359	2.271	1	.132	1.718	.850	3.471
	Dummy_SE_Middle	.924	.282	10.709	1	.001	2.520	1.449	4.383
	Dummy_SE_Bad	.912	.284	10.339	1	.001	2.490	1.428	4.341
	EnableNew	.085	.269	.100	1	.752	1.089	.642	1.846
	Received_TAPsEcig	.116	.224	.267	1	.605	1.123	.724	1.741
	Dummy_EN9_Middle	.247	.231	1.151	1	.283	1.281	.815	2.012
	Dummy_EN9_Cheap	.137	.446	.094	1	.760	1.146	.478	2.750
	Dummy_HH_Ecig	.142	.352	.164	1	.685	1.153	.579	2.297
	Dummy_HH_SMnotEcig	.552	.236	5.468	1	.019	1.736	1.093	2.757
	Dummy_friend_Ecig	.849	.266	10.164	1	.001	2.336	1.387	3.937
	Dummy_friend_SMnotEcig	1.329	.290	20.993	1	.000	3.776	2.139	6.666
	Dummy_IDOL_Ecig	.842	.618	1.856	1	.173	2.320	.691	7.789
	Dummy_IDOL_SMnotEcig	.014	.437	.001	1	.975	1.014	.431	2.386

a. The reference category is: ไม่ใช้/ไม่สูบ.

© 2023 Benjakul S. et al.
